# Facile One-Pot Synthesis of Hyperbranched Glycopolymers in Aqueous Solution via a Hydroxy/Cu(III) Redox Process

**DOI:** 10.3390/polym12092065

**Published:** 2020-09-11

**Authors:** Feng Liu, Yuangong Zhang, Xiaohui Hao, Qian Zhou, Ying Zheng, Libin Bai, Hailei Zhang

**Affiliations:** 1 College of Physics Science & Technology, Hebei University, Baoding 071002, China; liufeng@hbu.edu.cn (F.L.); haoxiaohui@hbu.edu.cn (X.H.); 2 College of Basic Medicine, Hebei University, Baoding 071002, China; 3 College of Chemistry and Environmental Science, Hebei University, Baoding 071002, China; 18131914973@163.com (Q.Z.); zyyzhengying@163.com (Y.Z.); zhonggou556@hbu.edu.cn (L.B.)

**Keywords:** hyperbranched glycopolymer, Cu(III), redox, amyloid fibrillation

## Abstract

In this study, a self-condensing vinyl copolymerization/redox (SCVP/Redox) system was constructed to prepare hyperbranched poly(methyl-6-*O*-methacryloyl-*α*-*D*-glucoside) by using Cu(III) as the initiator in aqueous solution, in which the –OH group in C-2, C-3 and C-4 position on pyranose rings could be initiated by Cu(III). The branched and linear units were clearly distinguished by nuclear magnetic resonance (^1^H NMR) to estimate the degree of branching (*DB*). When the ratio of Cu(III) to monomer fixed at 0.5:1, the *DB* value reached 0.32, which was higher than the product initiated by Ce(IV). Moreover, the inhibition activity of the products on amyloid fibrillation was investigated by using the hen egg-white lysozyme (HEWL) as a model based on the difference of the initiation sites. The results showed that the –OH groups in C-4 position might play an important role in this process.

## 1. Introduction

Glycopolymers, which are defined as the synthetic polymers bearing carbohydrates moieties as pendant or terminal groups, have drawn considerable attention in the fields of biomedicine and biomaterials, especially for drug-delivery systems, macromolecular drugs, biocatalysts and tissue engineering [1,2,3,4]. In recent years, many researchers have devoted themselves into the syntheses of glycopolymers with unique topological structures. Although the synthesis of glycodendrimers was viewed to be time consuming and tedious, the exploration of glycodendrimers has still attracted wide attention and some dramatic enhancement in their properties and functionality can be observed [5,6]. Recently, a better alternative has been made accessible by developing hyperbranched glycopolymers via a one-pot reaction process. Compared to glycodendrimers, the hyperbranched glycopolymers possess similar physical and chemical properties.

It is an acceptable approach to use the monomer containing carbohydrate unit for hyperbranched glycopolymers polymerization. Narain and coworkers synthesized galactose-based hyperbranched glycopolymers via the reversible addition-fragmentation chain transfer (RAFT) polymerization. The redox-responsive galactose-based glycopolymers are composed of 2-lactobionamidoethylmethacrylamide and 2-aminoethylmethacrylamide [7]. Bai et al., developed a new type of supramolecular hyperbranched glycopolymers by exploring an AB_2_-type macromolecular monomer. The AB_2_-type macromolecular monomer consisted of one lithocholic acid and two *β*-cyclodextrin terminated poly(2-(dimethylamino)ethyl methacrylate) segments [8]. Pieters and coworkers introduced the alkyne groups on meta-nitrophenyl *α*-galactoside. The meta-nitrophenyl *α*-galactoside was linked to azide-containing hyperbranched polyglycerols via a copper-catalyzed alkyne-azide cycloaddition reaction to give the hyperbranched glycopolymer with high binding capacity [9]. Till to now, it still needs further study for hyperbranched glycopolymer polymerization under simple and mild reaction condition with low-cost [10,11].

The supernormal valence transition metals such as Cu(III), Ni(IV), and Ce(IV) can efficiently react with hydroxyl groups and then generate a free radical polymerization [12,13]. Hence, we developed a self-condensing vinyl copolymerization/Redox (SCVP/Redox) system to explore a new library of hyperbranched glycopolymers by using Ce(IV) as the initiator [14]. Although the hyperbranched glycopolymers can be obtained in one step without tedious protection-deprotection procedures, the product shows a higher inhibition activity upon amyloid fibrillation and lower degree of branching (*DB*) value than 0.18. In addition, over 80% –OH groups on the pyranose ring cannot be initiated in the Ce(IV)-based SCVP/Redox system. The –OH group in the C-4 position on the pyranose ring is difficult to be initiated by Ce(IV) and the activity difference among the three –OH groups cannot be accurately evaluated. Therefore, it is significant to develop a novel SCVP/Redox system for the following purposes: (1) improving the degree of branching (*DB*); (2) activating the –OH group in C-4 position and initiating the racial polymerization; (3) evaluating the activity difference among the three –OH groups.

In this study, Cu(III) was used to replace Ce(IV) to initiate the polymerization of hyperbranched poly(6-*O*-MMAGlc)s. 6-*O*-MMAGlc was employed as an AB_3_* type inimer, in which A group contains a double bond and B contains the hydroxyl group. Fortunately, the redox reaction could occur on the proton (H12) in the –OH group located at the C-4 position on pyranose ring, as well as the protons (H13 and H14) in the –OH groups located at the C-3 and C-2 positions. In addition, more –OH groups located at the C-3 and C-2 positions take part in the redox reaction. Thereby, the DB values of hyperbranched poly(6-*O*-MMAGlc)s with Cu(III) as initiator were higher than the DB value reported in previous study. The activity test suggested that the –OH group in the C-4 position on pyranose ring plays an important role on the inhibitory ability upon amyloid fibrillation.

## 2. Materials and Methods

### 2.1. Materials

Methyl *α*-*d*-glucoside (98%), vinyl methacrylate (>98.0%), thioflavin T (ThT, 98%), (methyl sulfoxide)-*d_6_* (DMSO-*d_6_*, 99.9 atom% D) and deuterium oxide (D_2_O, 99.9 atom% D) were obtained from J&K Chemicals (Beijing, China). Novozym 435 was purchased from Novozymes Biotech (Tianjin, China). Hen egg white lysozyme (HEWL, ≥90%) was received from Sigma-Aldrich St. Louis (Shanghai, China). Azodiisobutyronitrile (AIBN, 99%) was purchased from Sigma-Aldrich (Shanghai, China) and recrystallized before use. Distilled deionized water was prepared from a Millpore Filtration System (Millpore, Bedford, MA, USA). A high-precision and ready-to-use dialysis bag (Nominal 1000) was purchased from Shanghai Green Bird Science and Technology Development Co., Ltd. (Shanghai, China). The diperiodatocuprate(III) (Cu(III)) [15] and methyl 6-*O*-methacryloyl-*α*-*d*-glucoside (6-*O*-MMAGlc) [16] were synthesized according to the methods reported in the literature.

### 2.2. Instrumentation

The spectra of nuclear magnetic resonance (^1^H NMR) were performed on a Bruker AVIII 600 MHz (Bruker, Karlsruhe, Germany). Number-average molecular weight (*M_n_*), weight-average molecular weight (*M_w_*), polydispersity (*PDI*), and (*η*) were determined by gel permeation chromatography (GPC) (Malvern Instruments Ltd., Malvern, PA, USA) which was equipped with RI detector, two-angle light scattering detector and viscosity detector. The system was calibrated with polyethylene oxide std-PEO22K and eluted with 0.1 M NaNO_3_ solution with a flow rate of 1 mL·min^−1^ at 30 °C. The calibration was performed at 30 °C and a flow rate of 1 mL·min^−1^. The samples were dissolved in 0.1 M NaNO_3_ solution and passed through 0.2 μm filter before measurement.

### 2.3. Synthesis

Synthesis of Cu(III): the Cu(III) were synthesized according to the methods reported in the literature [15]. Copper sulphate (3.54 g), potassium metaperiodate (6.8 g), potassium persulphate (2.2 g) and KOH (9 g) were added in 250 mL water. The reaction was heated to boiling for 40 min, then concentrated to 100 mL and filtered through sintered glass crucible (G-4) to obtain the Cu(III) solution. The concentration of as-prepared Cu(III) solution was calculated based on the absorbance spectra.

**Scheme 1:**
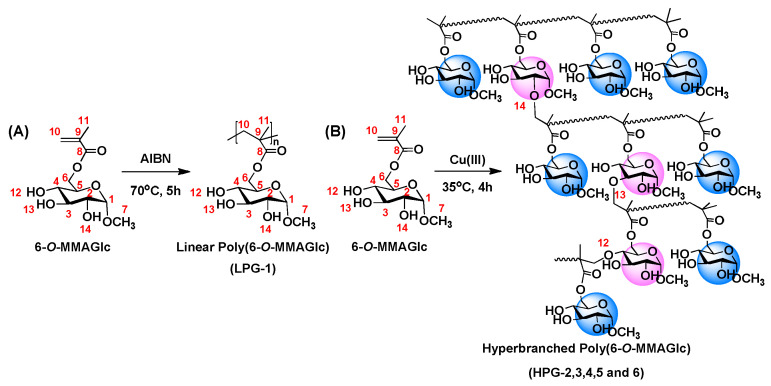
The synthesis of (**A**) linear poly(6-*O*-MMAGlc) and (**B**) hyperbranched poly(6-*O*-MMAGlc).

Synthesis of LPG-1: the synthetic route for the linear poly(6-*O*-MMAGlc) is shown in Scheme 1A. Mehtyl-6-*O*-methacryloyl-*α*-*d*-glucoside (300 mg, 1.15 mmol) was dissolved in 12 mL DMSO, removing the oxygen with three vacuum-nitrogen times. Then, AIBN (6.3 mg, 0.04 mmol) was added. The reaction was heated at 70 °C for 5 h. The crude product was precipitated with a mass of acetone, washed three times by acetone. The linear poly(6-*O*-MMAGlc) was purified by dialysis with molecular weight cut-off as 1000 (nominal 1000). Finally, a white solid (LPG-1) was obtained with freeze-dried. ^1^H NMR (600 MHz, DMSO-*d_6_*) δ 5.28-4.69 (m, 3H, H12-H14), 4.59 (s, 1H, H1), 4.19 (s, 1H, H6), 3.78 (s, 1H, H6), 3.56 (s, 1H, H5), 3.47–3.14 (m, 3H, H2, H3, H7), 3.03 (s, 1H, H4), 1.55 (s, 2H, H10), 1.20–0.53 (m, 3H, H11).

Synthesis of HPG-2,3,4,5 and 6: the synthetic route for the hyperbranched poly(6-*O*-MMAGlc)s was shown in Scheme 1B. In a typical experiment for HPG-2, 6-*O*-MMAGlc (300 mg, 1.15 mmol) was dissolved in 10.85 mL distilled deionized water. After nitrogen protection, the diperiodatocuprate (III) (DPC) (0.115 mmol, 1.15 mL) was added in the system, heated at 35 °C for 4 h. The filtrate solution was the raw hyperbranched poly(6-*O*-MMAGlc)s by suction filtration. Those were purified by dialysis with molecular weight cut-off as 1000 (Nominal 1000). Finally, **HPG-2** was freeze-dried from water and weighed to obtain the conversion. **HPG-2** (Figures S1 and S6): 1H NMR (600 MHz, DMSO-*d_6_*) δ 5.32–4.71 (m, 2.38H, H12-H14), 4.59 (s, 1H, H1), 4.18 (s, 1H, H6), 3.78 (s, 1H, H6), 3.54 (s, 1H, H5) 3.38–3.22 (s, 3H, H2, H3, H7) 3.02 (s, 1H, H4), 1.80 (s, 2H, H10), 0.87 (d, 3H, H11); **HPG-3** (Figure S2): 1H NMR (600 MHz, DMSO-*d_6_*) δ 5.32–4.71 (m, 2.26H, H12-H14), 4.58 (s, 1H, H1), 4.16 (s, 1H, H6), 3.79 (s, 1H, H6), 3.55 (s, 1H, H5), 3.37–3.20 (s, 3H, H2, H3, H7), 3.03 (s, 1H, H4), 1.78 (s, 2H, H10), 0.87 (d, 3H, H11); **HPG-4** (Figure S3): 1H NMR (600 MHz, DMSO-*d_6_*) δ 5.32–4.71 (m, 2.20H, H12-H14), 4.57 (s, 1H, H1), 4.16 (s, 1H, H6), 3.81 (s, 1H, H6), 3.56 (s, 1H, H5), 3.32–3.18 (s, 3H, H2, H3, H7), 3.02 (s, 1H, H4), 1.78 (s, 2H, H10), 0.84 (d, 3H, H11); **HPG-5** (Figure S4): 1H NMR (600 MHz, DMSO-*d_6_*) δ 5.32–4.71 (m, 2.10H, H12-H14), 4.58 (s, 1H, H1), 4.16 (s, 1H, H6), 3.79 (s, 1H, H6), 3.55 (s, 1H, H5), 3.34–3.18 (s, 3H, H2, H3, H7), 3.03 (s, 1H, H4), 1.74 (s, 2H, H10), 0.89 (m, 3H, H11); **HPG-6**: 1H NMR (600 MHz, DMSO-*d_6_*) δ 5.32–4.71 (m, 2.05H, H12-H14), 4.58 (s, 1H, H1), 4.16 (s, 1H, H6), 3.88 (d, 1H, H6), 3.55 (s, 1H, H5), 3.47–3.18 (m, 3H, H2, H3, H7), 3.02 (s, 1H, H4), 1.77 (s, 2H, H10), 0.89 (m, 3H, H11).

### 2.4. Cell Viability Assay

Methyl thiazolyl tetrazolium (MTT) assay was performed to examine the physical cytotoxicity of the sample, in which Hela (human cervical carcinoma) cell line was used. The cells supported by the Chinese Academy of Medical Sciences, Peking Union Medical College (Beijing, China). Hela cells (2 × 10^4^ cells/mL) were incubated at 37 °C with Dulbecco’s modified Eagle’s medium (DMEM) containing 10% FBS (fetal bovine serum) and antibiotic/anti-mycotic drugs under CO_2_ atmosphere. In this proposal, cells were placed in 96-well plates for 24 h with the addition of different concentrations of the products. Then the Hela cells were washed with phosphate-buffered saline (PBS) after the removing the medium and followed by incubated for 4 h with MTT solution (20 μL, 5 mg/mL). After removing the medium, 100 μL DMSO was added to each well. The absorbance of each well was determined by the ELX-800 microplate reader at 490 nm. The cell viability (%) was calculated based on the ratio of the number of surviving cells in test samples to control groups.

### 2.5. Amyloid Inhibition Test

#### 2.5.1. Lysozyme Sample Preparation

The hydrochloric acid (pH = 2.0) containing 140 mM NaCl and 0.01% (*w*/*v*) NaN_3_ was initially prepared, and dissolving lysozyme to prepare stock solution. Lysozyme solution with or without addition poly(6-*O*-MMAGlc) were incubated at 55 °C for 168 h without stirring. ThT fluorescence assay was used to investigate the formation of the fibril.

#### 2.5.2. Thioflavin T (ThT) Fluorescence Measurement

Thioflavin T (ThT) with accurate weighing was dissolved in phosphate buffer solution (pH = 7.4) to prepare the stock solution ([ThT] = 10 μM). 60 μL lysozyme sample solution was added into 2 mL ThT phosphate stock solution, and then mixed for 1 min. The fluorescence intensity at 486 nm was determined by F-7000 FL (Hitachi, Tokyo, Japan) a spectrophotometer with the excitation wavelength of 440 nm in the quartz cuvette. The excitation and emission slits were set to 20 nm and 10 nm, respectively. The inhibition ratio (C) was calculated by the following equation:

(1) $$C=\frac{I_{0}-I}{I_{0}}$$

where *I*_0_ and *I* were the ThT fluorescence intensity without and with samples, respectively.

## 3. Results

### 3.1. Nuclear Magnetic Resonance (NMR) Spectra of Polymers

In previous study, we synthesized the hyperbranched glycopolymers initiated by Ce(IV) in DMSO solution. The ^1^H NMR analyses showed that the oxygen radical (C–O) was generated by the redox reaction of Ce(IV) with –OH groups on the pyranose, while the pyranose rings were preserved [14]. Herein, we tried to construct a SCVP/Redox system in aqueous solution. Fortunately, the pyranose units and the ester bonds can be preserved in the presence of Cu(III). The alkaline environment may be favorable for preserving the pyranose rings and ester bonds in the monomer.

**Figure 1:**
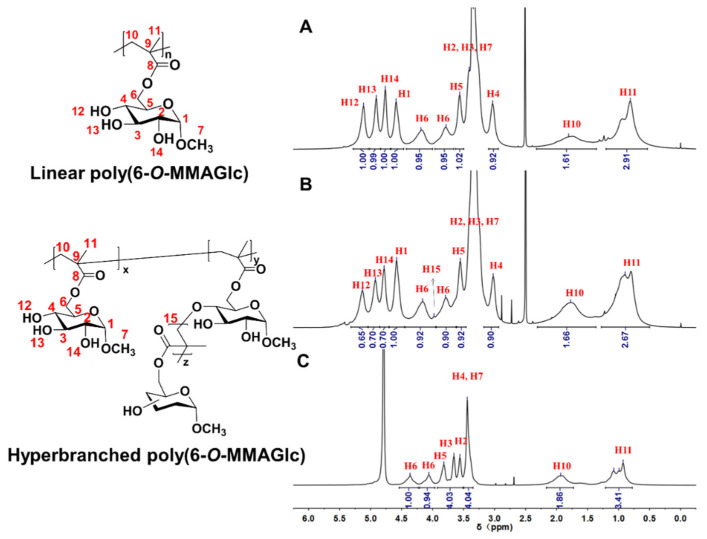
Nuclear magnetic resonance (^1^H NMR) spectra of LPG-1 and HPG-6 ((**A**) LPG-1 in methyl sulfoxide-*d_6_* (DMSO-*d_6_*); (**B**) HPG-6 in DMSO-*d_6_*; (**C**) HPG-6 in D_2_O).

Then Cu(III) was used as initiator to construct a SCVP/Redox system in aqueous solution and 6-*O*-MMAGlc was served as an AB_3_*-type inimer. In addition, a linear analogue LPG-1 was synthesized as a control. The ^1^H NMR spectrum of LPG-1 (Figure 1A) clearly displayed the peaks of the protons in –OH groups and pyranose rings. Compared with 6-*O*-MMAGlc [14], the integral area and chemical shift of protons in –OH groups were unchanged, implying the hydroxyl groups were preserved in radical polymerization process. HPG-6 was prepared by using Cu(III) as the initiator in which the feed ratio was 0.5 ([Cu(III)]_0_/[6-*O*-MMAGlc]_0_). For HPG-6, the peaks of H1, H6, H5, H3, H7, H2, H4, H10 and H11 located at 4.58, 4.16(3.79), 3.55, 3.35, 3.35, 3.35, 3.02, 1.77 and 0.89 ppm in the ^1^H NMR spectrum (Figure 1B), respectively. This result indicated that the C–C bond in the methylene group was not cleaved and the pyranose rings were retained. The chemical shifts of HPG-6 shown in the ^1^H NMR spectrum (Figure 1B) was similar with LPG-1, while the peak integrals of hydroxyl groups (H12, H13 and H14) significantly decreased. The results indicated that the hydroxyl groups were participated in radical polymerization process. For a linear analogue, the integral ratio of H12:H13:H14 is 1:1:1 and the value for HPG-6 was determined as 0.65:0.70:0.70. The degree of branching can be calculated based on the ^1^H NMR results by referring to a previous study.

(2) $$DB=\frac{BP}{D_{\max}}=\frac{3A_{H1}-\left(A_{H12}+A_{H13}+A_{H14}\right)}{3A_{H1}}$$

where AH1, AH12, AH13 and AH14 represent the integral areas of the H1, H12, H13 and H14 protons in 6-*O*-MMAGlc. For the linear product LPG-1, the area ratio of H12, H13, H14 and H1 is determined to be 1:1:1:1. Therefore, *DB* value of LPG-1 is calculated to be zero. The *DB* values of HPG-2, HPG-3, HPG-4, HPG-5 and HPG-6 are calculated to be 0.21, 0.25, 0.27, 0.30 and 0.32.

**Figure 2:**
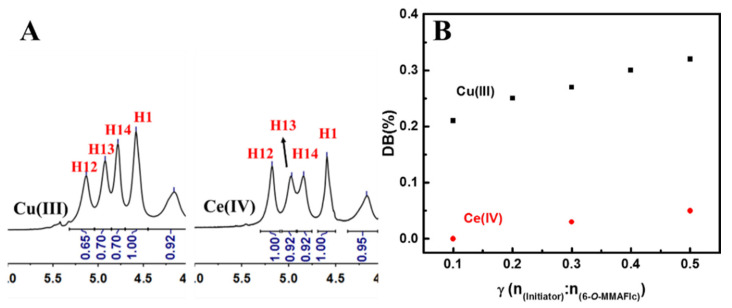
(**A**) The ^1^H NMR spectra of glycopolymers initiated by Cu(III) and Ce(IV); (**B**) The curve of the feed ratio (γ) vs. degree of branching (DB).

In our previous works, the hyperbranched poly(6-*O*-MMAGlc)s were prepared by using Ce(IV) as an initiator and the integral ratio of H12:H13:H14 is 1:0.92:0.92 when the feed ratio is 0.5:1 ([Ce(IV)]_0_/[6-*O*-MMAGlc]_0_). It should be noted the H12 in the –OH group located at the C-4 position in pyranose ring cannot be initiated in the Ce(IV)-based SCVP/Redox system. The initiation behavior only occurred on the protons (H13 and H14) in the –OH groups which are located at the C-3 and C-2 positions in pyranose rings. As for the hyperbranched poly(6-*O*-MMAGlc)s initiated by Cu(III), 35% H12 protons in the –OH group located at the C-4 position can be initiated when the feed ratio is also 0.5:1 ([Cu(III)]_0_/[6-*O*-MMAGlc]_0_). On the other hand, 30% H13 and H14 protons were initiated in this system, which is higher than the percent (8%) in Ce(IV)-based SCVP/Redox system (Figure 2). As a result, the hyperbranched poly(6-*O*-MMAGlc)s initiated by Cu(III) show the higher value than that of the products in the Ce(IV)-based SCVP/Redox system (0.32 vs. 0.07). In aqueous solution, Cu(III) may possesses a higher activity in the SCVP/Redox system. 

### 3.2. Gel Permeation Chromatography (GPC) Test for Polymers

To further investigate the effect of the feed ratio (γ) on *DB* value, molecular weight, Mark–Houwink exponent (*α*) and yield, the products were measured with multi-detector gel permeation chromatography (GPC) equipped with a multi-angle laser light-scattering (MALLS) detector (Figure S5). As increasing the initial concentration of Cu(III), the PDI increased from 1.6 to 2.8 and the *M_w_* decreased from 5.7 × 10^4^ to 2.8 × 10^4^ g/mol (details shown in Table 1). The addition of Cu(III) enhances the initiation rate and forms more initiation centers, which give rise to the decrease in molecular weight. Meanwhile, more branching points also be generated as the addition of Cu(III), resulting in the higher *DB* values.

Generally, the *α* parameter of linear polymers usually ranged from 0.6 to 0.8, while the *α* parameter of the hyperbranched polymers always less than 0.5 [17,18]. Furthermore, the low *α* parameter is corresponding to the high level of *DB*. The glycopolymer (LPG-1) initiated by AIBN showed an *α* value of 0.60, suggesting that LPG-1 was a linear structure. The decreasing of *α* value from 0.52, 0.50, 0.49, 0.48 to 0.45 for HPG-2, HPG-3, HPG-4, HPG-5 and HPG-6, were consistent with increasing of *DB* (0.21, 0.25, 0.27, 0.30 to 0.32) determined by the ^1^H NMR data. The polymer LPG-1 and polymer HPG-2 own similar *M*_n_ with different topological structures. Therefore, HPG-2 was selected for the further studies.

### 3.3. Inhibitory Activity of Polymers

**Figure 3:**
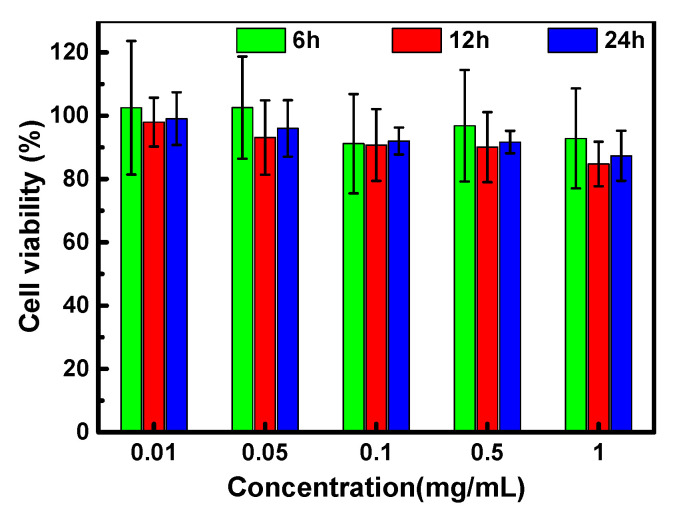
In vitro viability of human cervical carcinoma (Hela) cells at different concentrations of HPG-2 incubated for 6, 12 and 24 h.

The growth inhibitory activity of HPG-2 against Hela cells is shown in Figure 3. The result showed that the prepared glycopolymers own a low cytotoxicity (over 90% cell viability) within 24 h of incubation time, in which the concentrations of HPG-2 were ranging from 0.01 to 1.0 mg/mL. Due to the low cytotoxicity, the prepared glycopolymers show a promising application in biomedical and biomaterials fields.

**Figure 4:**
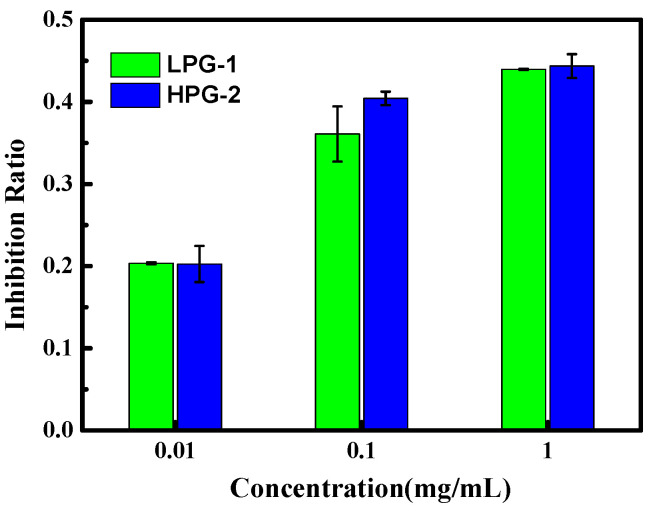
The inhibition ratio of LPG-1 and HPG-2 on the fibrillation of hen egg-white lysozyme (HEWL).

**Figure 5:**
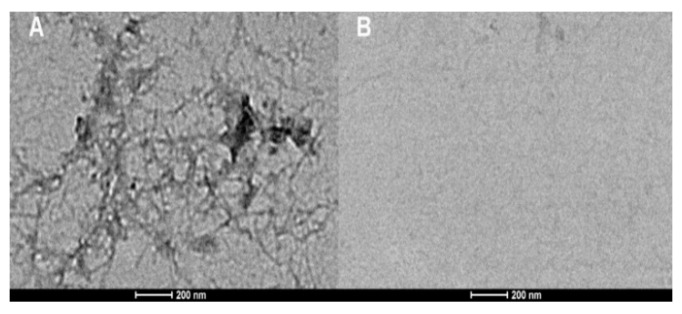
Electron microscopy analysis of HEWL incubated without or with glycopolymer. (**A**) Transmission electron microscopy (TEM) image of HEWL alone; (**B**) TEM image of HPG-2 and HEWL.

The inhibition activity of the product on amyloid fibrillation was examined and the hen egg-white lysozyme (HEWL) was chosen as a model. HEWL was incubated at 55 °C for 7 days in the absence or presence of products. The inhibition ratios were calculated by the ThT fluorescence assay in Figure 4. The results showed that HPG-2 has similar inhibition activity to LPG-1 and the inhibition activity shows a positive relationship with the concentration. The transmission electron microscopy (TEM) image (Figure 5A) indicated typical fibrillation in the control runs. The non-aggregation behavior against HEWL fibrillation was observed for HPG-2 depicted in Figure 5B. The TEM results match well with the ThT assay.

HPG-2 contains fewer hydroxyl groups than LPG-1, while the inhibition ratio of HPG-2 was similar to that of the linear analogue LPG-1. In general, the consuming of the hydroxyl groups might lead to the decrease or loss of biological activity. Based on the polymerization mechanism, the hydroxyl groups were sacrificed to form the initiating centers or branching points. Thus, the biological activity of HPG-2 might be lower than LPG-1 due to the loss of –OH groups. Interestingly, the prepared hyperbranched glycopolymer showed a similar inhibition activity on amyloid fibrillation compared with that of linear analogue. It may be caused by the “cluster effect” of the branched architecture [19,20]. In the previous study, the hyperbranched glycopolymer prepared by Ce(IV) showed a higher inhibition activity than that of linear analogue. The difference in the inhibition activity between two different kinds of hyperbranched glycopolymer may be owing to the different initiation sites. According to the NMR results, the redox reaction mainly occurred on the H13 and H14 in the Ce(IV)-based SCVP/Redox system. As for HPG-2 initiated by Cu(III), the proton H12 on C-4 positions can also be initiated in the Cu(III)-based SCVP/Redox system. But HPG-2 showed a lower inhibition activity as compared to the corresponding product in the previous study. The results suggested that H12, the proton in the –OH group in the C-4 position, might play an important role in inhibiting amyloid fibrillation.

## 4. Conclusions

In summary, a new type of hyperbranched glycopolymers was synthesized in aqueous solution by SCVP/Redox strategy with using Cu(III) as the initiator. The formation of a branched structure was confirmed by the multi-detector GPC analysis and ^1^H NMR. When increasing the amounts of Cu(III), the DB increased and molecular weights decreased. The product showed a low cytotoxicity. The amyloid inhibitory activity tests showed that the –OH group in the C-4 position, might play an important role in inhibiting amyloid fibrillation. We hope that these investigations can offer a versatile approach to develop a new library of hyperbranched glycopolymers and explore their potential applications in biomedicine and biomaterials.

## Figures and Tables

**Table 1:** The detailed parameters of the glycopolymers obtained.

Sample	γ ^a^	Yield (%)	*M*_n_^b^ (10^4^ g/mol)	*M*_w_^b^ (10^4^ g/mol)	PDI (*M*_w_/*M*_n_)	α ^c^	*DB* ^d^
LPG-1	-	67	3.3	4.5	1.36	0.6	0
HPG-2	0.1	35	3.4	5.7	1.67	0.52	0.21
HPG-3	0.2	33	3.1	5.5	1.77	0.50	0.25
HPG-4	0.3	35	2.8	4.8	1.71	0.49	0.27
HPG-5	0.4	30	1.8	3.6	1.97	0.48	0.30
HPG-6	0.5	29	1.0	2.8	2.87	0.45	0.32

^a^ the radio of [Cu(III)]:[6-*O*-MMAGlc]; ^b^ calculated by gel permeation chromatography (GPC); ^c^ calculated by plotting log intrinsic viscosity to log *M_w_*; ^d^ calculated by the ^1^H NMR data.

## Supplementary Materials

The following are available online at https://www.mdpi.com/2073-4360/12/9/2065/s1: Figure S1: The 1H NMR spectrum of HPG-2 measured in DMSO-d6., Figure S2: The 1H NMR spectrum of HPG-3 measured in DMSO-d6, Figure S3: The 1H NMR spectrum of HPG-4 measured in DMSO-d6, Figure S4: The 1H NMR spectrum of HPG-5 measured in DMSO-d6, Figure S5: The GPC curves of LPG-1 and HPG-2, Figure S6: The 1H NMR spectrum of HPG-2 measured in DMSO-d6.
