# Supplementary material for: Facile One-Pot Synthesis of Hyperbranched Glycopolymers in Aqueous Solution via a Hydroxy/Cu(III) Redox Process
Source: Polymers (Basel). 2020 Sep 11;12(9):2065. doi: 10.3390/polym12092065 (PMC7570359; doi:10.3390/polym12092065)
Supplement: Supplementary file 1 [file polymers-12-02065-s001.pdf]

polymers

MDPI

# Supplementary Files of Facile One-Pot Synthesis of Hyperbranched Glycopolymers in Aqueous Solution via a Hydroxy/Cu (III) Redox Process

**Feng Liu[1], Yuangong Zhang[3,*], Xiaohui, Hao[1], Qian Zhou[2], Ying Zheng[2], Libin Bai[2], and Hailei Zhang[2,*]**

**Contents**

*1. Nuclear Magnetic Resonance (NMR) of the Hyperbranched Glycopolymers*

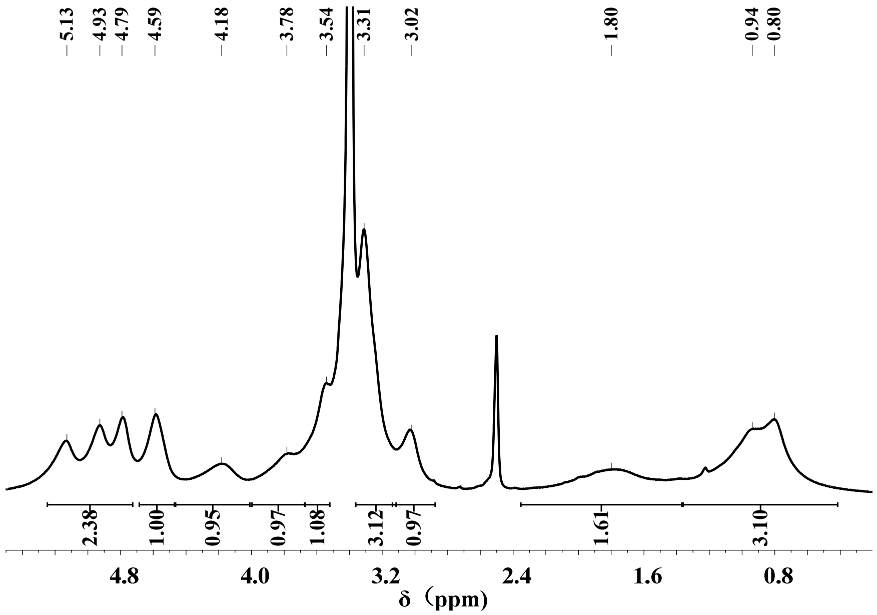


**Figure S1.** The nuclear magnetic resonance ($^1$H NMR) spectrum of **HPG-2** measured in (methyl sulfoxide)-$d_6$ (DMSO-$d_6$).

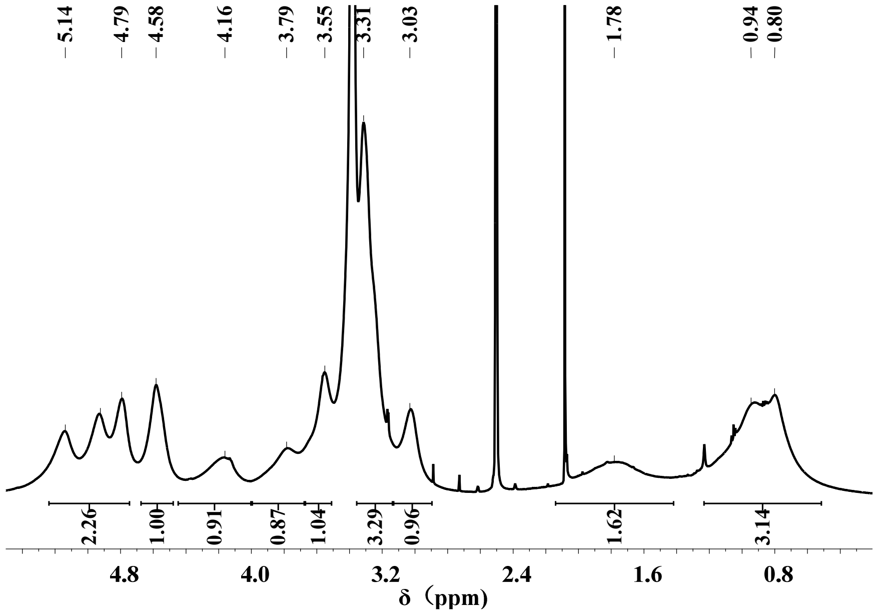

**Figure S2.** The $^{1}$H NMR spectrum of **HPG-3** measured in DMSO-*d*6.

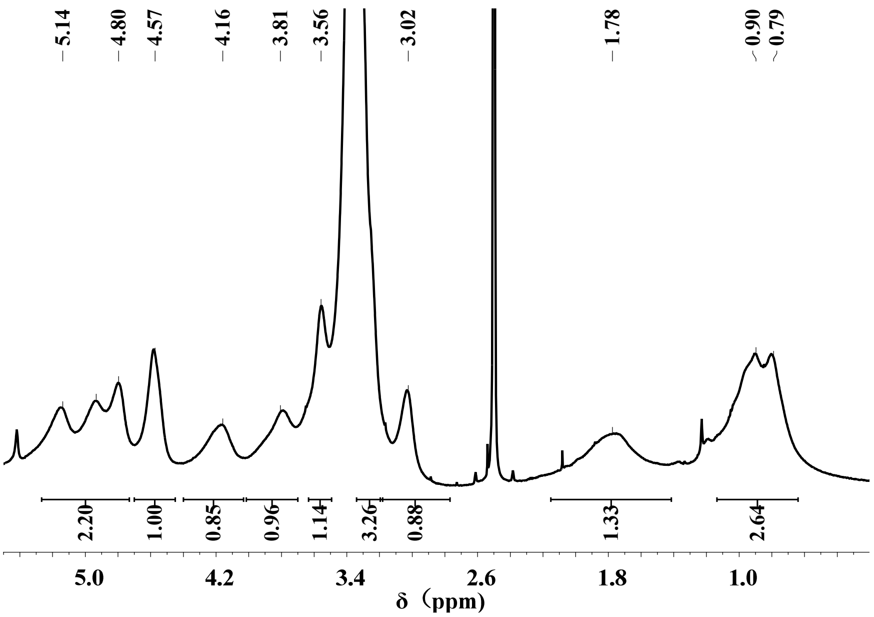


**Figure S3.** The $^{1}$H NMR spectrum of **HPG-4** measured in DMSO-*d*6.

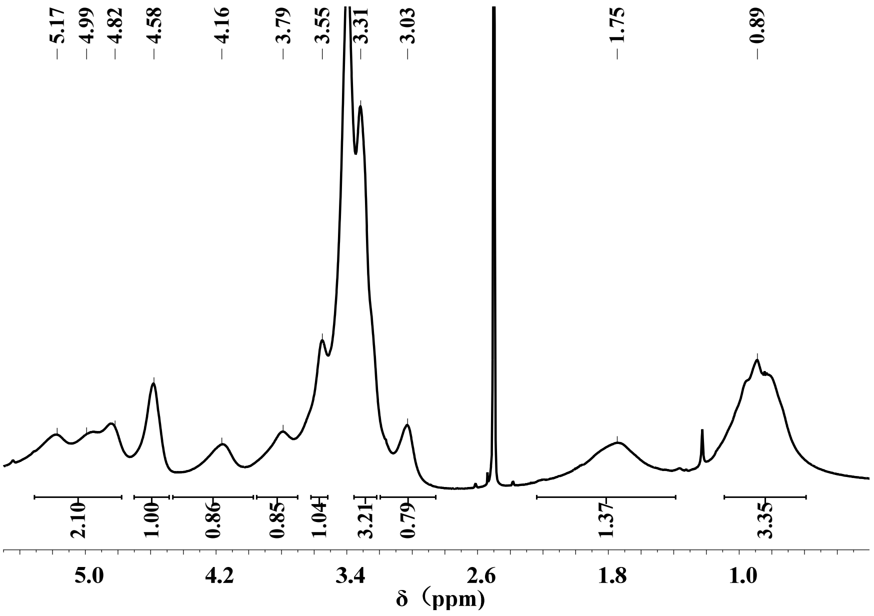


**Figure S4.** The $^{1}$H NMR spectrum of **HPG-5** measured in DMSO-*d*6

## *2. GPC curves of LPG-1 and HPG-2*

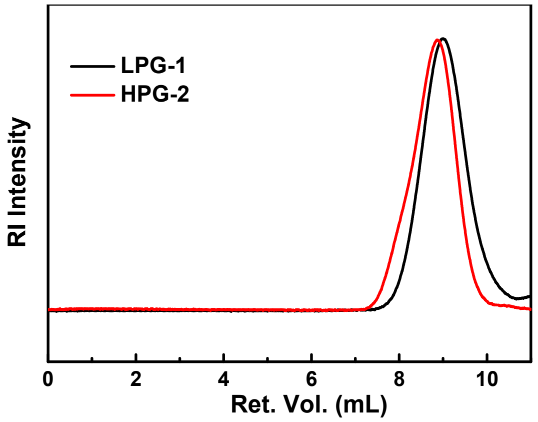


**Figure S5.** The gel permeation chromatography (GPC) curves of **LPG-1** and **HPG-2.**

## *3. Elimination of the Possibility of Such Side Oxidation Reactions*

Figure S6 showed the 1H NMR spectrum of HPG-2 up to 11 ppm. It clearly to see from the NMR spectra that there is no signal of an aldehyde up to 11 ppm. In addition, we also performed a control experiment treating mehtyl-6-O-methacryloyl-$\alpha$-D-glucoside, a monomer having no acylate moiety, with Cu(III) at 70 °C for 5 h. Then the residue was collected and monitored by thin-layer chromatography (TLC) under iodine vapor (Rf=0.2, eluent agent: ethyl acetate/ methanol: 5/1), which is in accordance with that of mehtyl-6-O-methacryloyl-$\alpha$-D-glucoside. No other impurities were found based on the TLC monitor. The results indicate that the structure of the residue was unchanged, which eliminate the possibility of such side oxidation reactions.

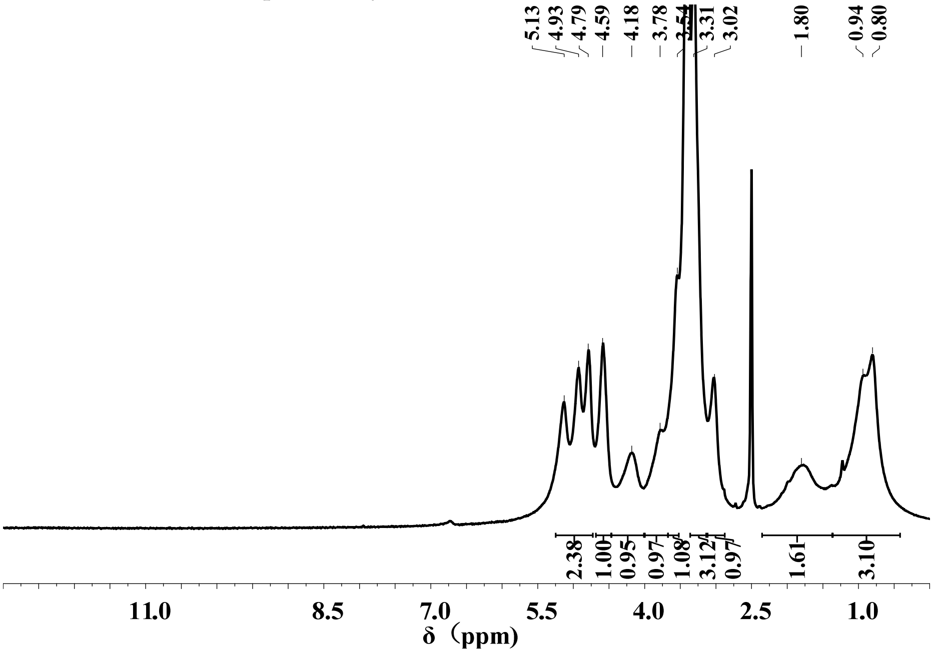


**Figure S6.** The $^1$H NMR spectrum of **HPG-2** measured in DMSO-*d*$_6$.

© 2020 by the authors. Licensee MDPI, Basel, Switzerland. This article is an open access article distributed under the terms and conditions of the Creative Commons Attribution (CC BY) license (http://creativecommons.org/licenses/by/4.0/).
